# Hydrocéphalie à pression normale

**DOI:** 10.11604/pamj.2014.18.241.4812

**Published:** 2014-07-23

**Authors:** Brahim El Jebbouri, Brahim El Mostarchid

**Affiliations:** 1 Service de Neurochirurgie, Hôpital Militaire d'Instruction Mohamed V, Rabat, Maroc

**Keywords:** hydrocéphalie, pression normale, DVP, hydrocephalus, normal pressure, DVP

## Image en medicine

Nous rapportons ici le cas d'un patient de 62 ans admis dans notre service pour troubles de la marche à type d'abasie, troubles intellectuels sous forme de défaut de concentration et désintérêt en plus de d'une incontinence urinaire évoluant depuis plus d'un an. Cette triade est évocatrice d'une hydrocéphalie à pression normale. Un scanner à été réalisé confirmant notre diagnostique montrant la dilatation ventriculaire sans effacement des sillions (A). Devant l'amélioration neuropsychique de notre patient suite à la réalisation de trois ponctions lombaire de déplétion, on a réalisé une dérivation ventriculo-péritonéale (B) et le patient est actuellement asymptomatique après un an du geste chirurgical.

**Figure 1:**
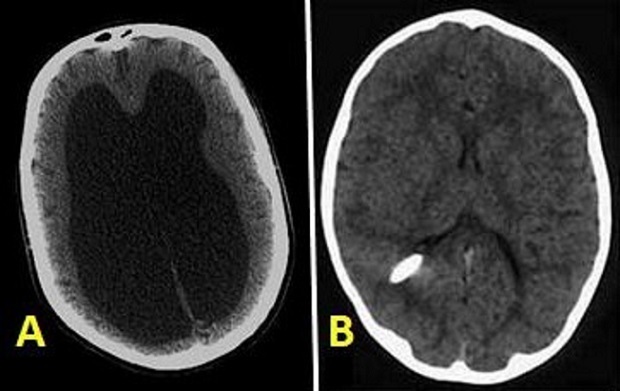
A) coupe axiale d'un scanner cérébral montrant une ventriculomégalie sans effacement des sillons corticaux. B) Coupe axiale d'un scanner cérébral après une dérivation ventriculo péritonéale montrant un retour à la normale du cerveau

